# Serum Vitamin A Levels May Affect the Severity of Ocular Graft-versus-Host Disease

**DOI:** 10.3389/fmed.2017.00067

**Published:** 2017-06-22

**Authors:** Jiefeng Tong, Renjian Hu, Yingying Zhao, Yang Xu, Xiaoying Zhao, Xiuming Jin

**Affiliations:** ^1^Hematology Department, Second Affiliated Hospital, School of Medicine, Zhejiang University, Hangzhou, China; ^2^Eye Center, Second Affiliated Hospital, School of Medicine, Zhejiang University, Hangzhou, China

**Keywords:** allogeneic hematopoietic stem cell transplantation, vitamin A, graft-versus-host disease, dry eye, cornea perforation

## Abstract

Allogeneic hematopoietic stem cell transplantation (HSCT) is a well-established therapeutic option for a range of inherited and acquired hematological disorders. However, graft-versus-host disease (GVHD) remains the leading cause of non-relapse mortality in allogeneic HSCT recipients. Ocular involvement occurs in up to 80% of chronic GVHD patients. In our cases, the diagnosis of vitamin A deficiency was suspected for GVHD patients. Serum vitamin A measurements were conducted to confirm clinical suspicions. Our study revealed significant decrease in serum levels of vitamin A in chronic liver GVHD patients. Although there have been many studies evaluating ocular manifestations in patients with GVHD, the present study is, to our knowledge, the first to study the relationship between vitamin A and ocular manifestations of GVHD in humans. Our data suggest that vitamin A deficiency affects the severity of ocular GVHD in adults.

## Introduction

Graft-versus-host disease (GVHD) is one of the most devastating long-term complications of allogeneic hematopoietic stem cell transplantation (HSCT) (1). Allogeneic HSCT is a well-established therapeutic option for a range of inherited and acquired hematological disorders (2). However, allogeneic HSCT can give rise to GVHD, either in acute or chronic form, which represents the major cause of morbidity and mortality after allogeneic HSCT (3). Chronic GVHD (cGVHD) is a very common long-term complication that occurs in 25–70% of patients (4). Unlike acute GVHD that affects mainly skin, liver, and gastrointestinal tract, cGVHD can also occur in other sites including eyes, mouth, musculoskeletal system, and lungs (5). Ocular manifestations may occur in up to 80% of cGVHD patients (6). Theoretically, cGVHD can affect any ocular structure, but it generally involves the ocular surface system such as cornea, conjunctiva, eyelids, meibomian glands, lacrimal, and its drainage system (6). In severe cases, ocular GVHD may lead to sight-threatening ocular surface complications like corneal ulceration and perforation (7).

Vitamin A is a fat-soluble vitamin ingested in the diet in two forms: as the retinol itself or as the provitamin carotene (8). Retinol is necessary for epithelial cell RNA and glycoprotein synthesis on the ocular surface. Therefore, vitamin A deficiency has a wide range of ocular manifestations, including conjunctival and corneal xerosis, keratomalacia, retinopathy, visual loss, and nyctalopia (8). Although the relationship between vitamin A deficiency and ocular complications is often reported in infants and children, whether vitamin A deficiency also leads to the development of ocular disease in adults is uncertain.

When cGVHD occurs in the liver, serum levels of vitamin A may be also significantly reduced because of abnormal metabolism of vitamin A. However, the association between vitamin A and ocular GVHD has not been established. Therefore, we performed this study in order to investigate the association between ocular manifestations of GVHD and serum levels of vitamin A. Our results suggest that vitamin A deficiency may affect the severity of ocular GVHD.

## Patients and Methods

This study was approved by the Ethics Committee of the Second Affiliated Hospital, Zhejiang University School of Medicine in China, complied with the tenets of the Declaration of Helsinki for research involving human tissue, and adhered to the ARVO statement on human subjects. Written informed consents were obtained from all patients after they received an explanation of the nature and possible consequences of the procedures.

Patients receiving allogeneic HSCT between January, 2013 and April, 2015 were included in this study. The inclusion criteria required patients to be older than 18 years of age. Details from each patient were recorded, including (1) disease history, (2) serum levels of vitamin A evaluated before HSCT, 3 months after HSCT, and during graft rejection by HPLC, (3) ocular examination findings by slit-lamp biomicroscopy, (4) dry eye symptoms and dry eye-related examination, and (5) treatment strategy and prognosis.

The exclusion criteria included the patients who suffered from systemic diseases related to dry eye, such as diabetes mellitus, Sjogren’s syndrome, or Stevens–Johnson syndrome, and the patients suffering from allergic conjunctivitis, herpes simplex virus keratitis, and glaucoma. All participants were free of cancer, liver disease, renal disease, stroke, transient cerebral ischemia, myocardial infarction, peptic ulcers, and gout. The exclusion criteria also required patients to have no acute GVHD.

### Eye Examinations

The serum levels of vitamin A were blind to eye doctors. The patients were evaluated through a slit lamp examination, and dry eye tests were performed as in our previous study, using the subjective symptom score, the non-invasive tear breakup time test (NITBUT), corneal fluorescein staining (CFS), and Schirmer’s *I* test (SIt) (9). The examination process was completed by the same doctor, and the results were recorded.

#### Schirmer’s *I* Test

The SIt employed the following procedure. After the application of a topical anesthesia, a filter paper strip (35 mm × 5 mm) was put into the lower eyelid conjunctival sac, and the lengths of the wetted parts were recorded after 5 min; lengths shorter than 10 mm were considered abnormal.

#### Non-Invasive Tear Breakup Time Test

The NITBUT was used to measure tear film stability. The patient was asked to close his or her eyes after fluorescein staining, and the time was measured until the first tear film rupture spot appeared. The average value was recorded from three measurements, and a score higher than 10 s was considered abnormal.

#### Fluorescein and Lissamine Green Staining

For fluorescein and lissamine green staining (CFS), the cornea was equally divided into the upper, middle, and lower three quadrants, the temporal and nasal bulbar conjunctiva was also divided into upper and lower parts, and the score of each part was recorded after staining as follows: 0 = no punctate staining, 1 = less than half stained, 2 = more than half stained, 3 = entirely stained. A cumulative score for each quadrant was given (0–9).

### Dry Eye Symptoms

We used a modified questionnaire (Table 1) based on previous reports (10), which was also used in our previous studies (9, 11). The results were obtained from the questionnaire survey, and items regarding foreign body sensation, photophobia, itching, aching, dryness, heavy sensation, blurred vision, fatigue, discomfort, ocular discharge, and lacrimation were listed. Each item, according to the frequency of occurrence, was rated from 0 to 3 as follows: 0 = no symptoms, 1 = occasional symptoms, 2 = mild intermittent symptoms, and 3 = recurring obvious symptoms, with a possible total of 0–33.

**Table 1:** **Dry eye questionnaire**.

Symptom	No	Occasionally	Incontinuous	Continuous
Foreign bodies sensation	0	1	2	3
Photophobia	0	1	2	3
Itching	0	1	2	3
Aching	0	1	2	3
Dryness	0	1	2	3
Heavy sensation	0	1	2	3
Blurred vision	0	1	2	3
Fatigue	0	1	2	3
Discomfortableness	0	1	2	3
Ocular discharge	0	1	2	3
Lacrimation	0	1	2	3

### Diagnosis of cGVHD

The diagnoses were based on either diagnostic clinical signs of cGVHD or required confirmation by histology as described by the NIH consensus (5).

### Grade of Liver cGVHD

The liver cGVHD was scored according to 2014 NIH consensus criteria: score 0, normal total bilirubin and alanine aminotransferase (ALT) and alkaline phosphatase (AP) <3 × normal upper limit (ULN); score 1, normal total bilirubin with ALT 3–5 × ULN or AP ≥3 × ULN; score 2, elevated total bilirubin but <3 mg/dL or ALT > 5 × ULN; score 3, elevated total bilirubin >3 mg/dL (12).

### Grade of Ocular GVHD

The diagnosis of ocular GVHD was based on newly developed dry eye with a mean Schirmer’s score of ≤5 mm or with a Schirmer’s score of 6–10 mm occurring in a patient with cGVHD in at least one other organ system (12). To evaluate the grade of GVHD, we used a modified criterion based on previous reports (13, 14) and our clinical experience. The scores of the ocular GVHD were as follows: grade 0 = no clinical signs of dry eye, grade 1 = dry eye without positive CFS, grade 2 = dry eye with positive CFS, grade 3 = dry eye with positive CFS and conjunctiva Lissamine Green staining, and grade 4 = dry eye with corneal ulcer.

### Serum Levels of Vitamin A

In order to detect the change in vitamin A levels caused by GVHD, we collected the patients’ peripheral blood samples before HSCT, 3 months after HCT, and during cGVHD n. Sera were obtained in the absence of anticoagulant agents and stored at ≤−80°C. The serum levels of vitamin A were measured by the HPLC method according to the instructions provided by the manufacturer.

### Treatment of Ocular GVHD

When cGVHD developed, standard immunosuppressive therapy was initiated. Ocular GVHD patients also receive local treatments, such as anti-inflammatory (including tacrolimus, corticosteroids, etc.), preservative-free eye drops, and vitamin A ointment (when vitamin A deficiency is found).

### Statistical Analysis

The statistical significance of correlations was determined with a paired *t-*test and correlation analysis using SPSS version 19.0 (SPSS, Inc., Chicago, IL, USA). Differences were considered to be statistically significant when the *P* < 0.05.

## Results

The final analysis included 46 patients (25 men and 21 women) with a mean age of 34.4 ± 10.5 years (range: 20–58 years). Table 2 shows the baseline characteristics of these patients, of which 19 were diagnosed as chronic cGVHD. The average time of cGVHD onset was 4–17 months after HSCT. The affected organs included the skin, liver, kidneys, eyes, gastrointestinal system, oral mucosa, and lungs. More than half of the cGVHD patients had more than two affected organs. Nine of these patients developed heavy, severe ocular manifestations of cGVHD, such as corneal ulcerations and cataracts. Among these nine patients, seven of them also experienced liver cGVHD and three (three eyes) suffered cornea perforation.

**Table 2:** **Characteristics of patients receiving allogeneic hematopoietic stem cell transplantation**.

Variable	All data
Patients	46
Age (mean ± SD, years)	34.4 ± 10.5(20–58)
Gender (M/F)	25/21
Graft-versus-host disease (GVHD)/non-GVHD	19/27
Onset of GVHD (months)	18.9 ± 9.8 (4–17)
Severe ocular GVHD	9
Cornea perforation	5

**Figure 1:**
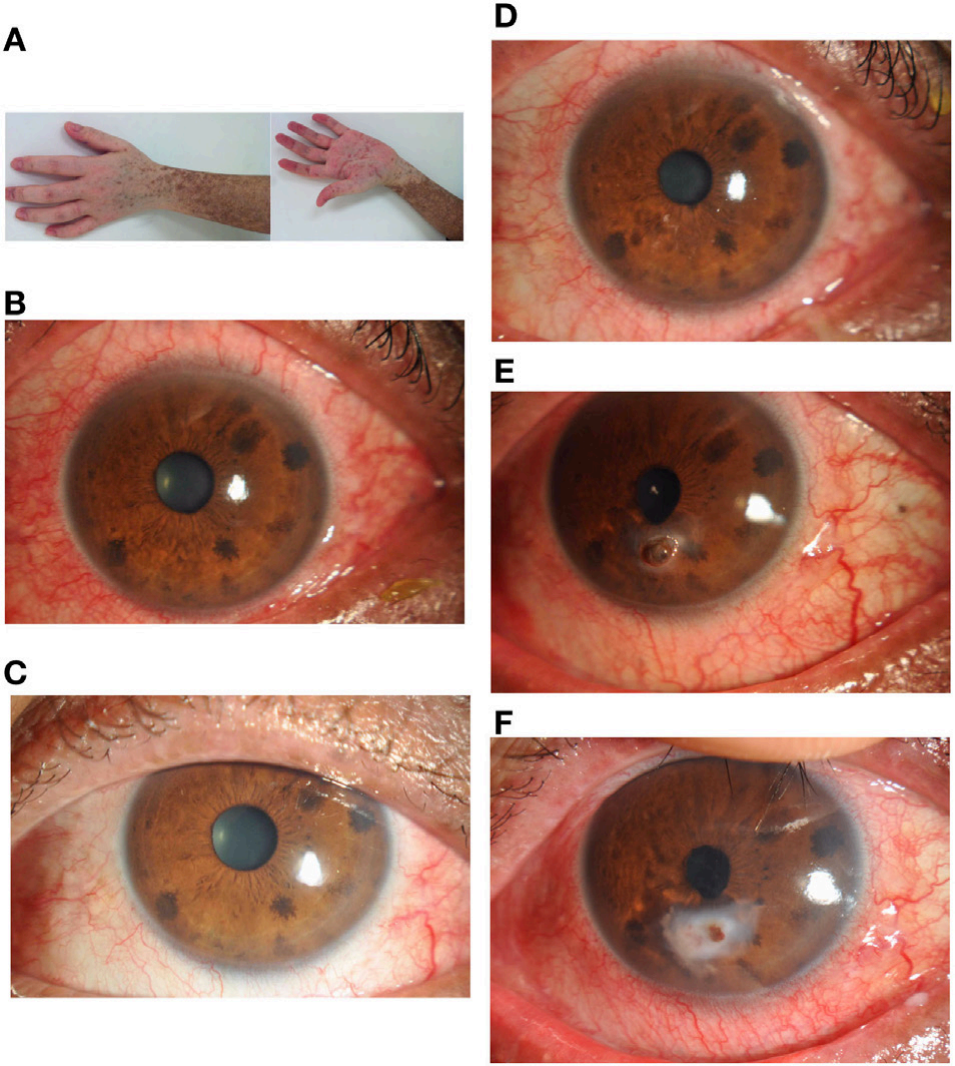
**A 28-year-old man developed chronic myeloid leukemia in 2003**. He underwent a 9/10 locus HLA-matched allogeneic bone marrow transplant in November 2013 with 0.512 μg/ml serum vitamin A. He described no ocular symptoms at 3 months after hematopoietic stem cell transplantation with 0.454 μg/ml serum vitamin A. Six months later, he developed chronic GVHD affecting the skin **(A)**, oral mucosa, gastrointestinal system, and eyes **(B)**. Ocular manifestations **(C)** were managed symptomatically with topical methylcellulose drops with 1.012 μg/ml serum vitamin A. In October 2014, he developed severe graft-versus-host disease affecting the skin, oral mucosa, liver, lungs, and eyes **(D)**. Ocular discomfort was managed symptomatically again with topical methylcellulose drops and artificial eye drops. One month later, he was referred to the ophthalmology department with a 2 mm perforation complicating a generalized progressive stromal melt **(E)** with 0.147 μg/ml serum vitamin A. Ocular perforation was managed with topical 0.1% flumetholon, 0.3 sodium hyaluronate drops, vitamin A gel, and a corneal bandage lens. The cornea perforation was healed **(F)** with 3 months treatment. At that time, the patient showed normal liver function and 0.527 μg/ml serum vitamin A.

There were no significant differences between GVHD patients and non-GVHD patients in baseline characteristics (Table 3). The vitamin A level in patients before HSCT was 0.709 ± 0.251 μg/ml (range from 0.354 to 1.221, normal over 0.3). Three months after HSCT, the average serum level of vitamin A was 0.718 ± 0.198 μg/ml (range from 0.389 to 1.220), which was not a significant change compared to the pre-levels (Table 4). When GVHD occurred, serum levels of vitamin A (0.965 ± 0.304 μg/ml) were slightly elevated compared with pre-levels (*t* = 2.553; *P* = 0.020). However, when liver GVHD persisted for more than 1 month, the level of vitamin A (0.18 ± 0.03) was significantly lower than at the onset of GVHD (*t* = 6.003; *P* = 0.004). Figure 1 shows the relationship between ocular GVHD and the fluctuation of serum levels of vitamin A after HSCT in a cGVHD patient.

**Table 3:** **Ocular graft-versus-host disease (GVHD) severity and serum vitamin A levels in patients with or without systemic GVHD**.

	Age	Dry eye syndromes	Ocular GVHD grade	The serum levels of vitamin A
GVHD	32.79 ± 10.40	9.05 ± 4.59	0.74 ± 0.56	0.71 ± 0.25
Non-GVHD	33.26 ± 7.68	7.30 ± 3.38	0.70 ± 0.54	0.71 ± 0.26
*t*	0.176	1.496	0.849	0.073
*P*	0.861	0.142	0.274	0.942

*P, the results of each stage after treatment and before treatment were compared, respectively*.

**Table 4:** **Compare the score of dry eye symptoms between graft-versus-host disease (GVHD) and non-GVHD patients**.

Serum vitamin A	Baseline (mean ± SD)	3 months	GVHD onset	1 month after liver GVHD	Paired difference (VS baseline)		
					3 months	GVHD onset	Liver GVHD
GVHD	0.71 ± 0.25	0.69 ± 0.17	0.97 ± 0.30	0.18 ± 0.03	*t* = 0.378, *P* = 0.710	*t* = 2.553, *P* = 0.020	*t* = 6.076, *P* = 0.004
Non-GVHD	0.71 ± 0.26	0.74 ± 0.22			*t* = 1.22, *P* = 0.233		

*P, the results of each stage after treatment and before treatment were compared, respectively*.

Table 5 shows that the average ocular GVHD grade of both eyes between GVHD and non-GVHD patients. Severe ocular GVHD always involved both eyes. The grade of ocular GVHD was significantly higher in patients with serum vitamin A deficiency. A strong correlation was found between serum vitamin A level and the grade of ocular GVHD (*r* = 0.830, *P* = 0.000). Ocular manifestations were improved in five patients with serum vitamin A level deficiency after treatment with vitamin A ointment. Figure 1 shows the change in ocular manifestations with changing serum levels of vitamin A in a cGVHD patient.

**Table 5:** **Compare the score of ocular graft-versus-host disease (GVHD) between GVHD and non-GVHD patients**.

Grade of ocular GVHD	Baseline (mean ± SD)	3 months	GVHD onset	1 month after liver GVHD	Paired difference		
					3-month VS baseline	GVHD (VS 3 months)	Liver GVHD VS
GVHD	0.74 ± 0.56	1.11 ± 0.66	1.42 ± 0.84	3.43 ± 0.98	*t* = 2.111, *P* = 0.049	*t* = 1.143, *P* = 0.268	*t* = 4.042, *P* = 0.007
Non-GVHD	0.70 ± 0.54	0.96 + 0.65			*t* = 2.563, *P* = 0.017		

## Discussion

Graft-versus-host disease is generally considered as an inflammatory process triggered by donor lymphocytes that recognize and destroy recipient tissues or organs. Innate immune cells and T cells from the donor have been identified in ocular specimens obtained from HSCT recipients, and alloreactive T cells were found predominantly in ocular lesions with cGVHD (15, 16). Moreover, cytokines and chemokines that promote T cell activation and proliferation were detected in the tears and ocular surface of patients with cGVHD (17, 18). However, it is not clear why only some cases of severe ocular GVHD progress to corneal epitheliopathy, ulceration, and perforation, the mechanism of which remains poorly understood. It has been shown that perforated corneal specimens resulting from cGVHD contain macrophages, T cells, apoptotic keratocytes and epithelial cells, and matrix metalloproteinase is highly expressed (7, 19–21). Chronic ocular inflammation can lead limbal stem cell deficiency or corneal neuropathy, and this disorder may also play a role in the pathogenesis of corneal ulceration and perforation in ocular GVHD (19–21). In this study, despite therapeutic intervention, three of the corneal ulcerations aggravated, resulting in one perforation and two severe ocular disorders; all of these patients suffered from liver cGVHD.

Retinol is transformed in the liver into retinoic acid, the active form that induces cell differentiation and modulates gene expression. Retinol is necessary for epithelial cell RNA and glycoprotein synthesis on the ocular surface. Vitamin A deficiency potentially affects all the epithelial cells of the eye. Disorders may range from simple dry eye up to xerosis, severe keratomalacia, corneal scarring, and perforation. Retinoic acid has recently been shown to control the phenotype and extracellular matrix composition of corneal stromal cells cultured *in vitro* as monolayers (22). In this study, the diagnosis of vitamin A deficiency was suspected in GVHD patients; therefore, measurement of serum vitamin A was conducted to confirm clinical hypothesis.

The ocular manifestations of vitamin A deficiency are variable. In cases of GVHD, the serum levels of vitamin A were slightly elevated compared with the pre-levels. The results suggest that the serum levels of vitamin A may be elevated under stress or disease states. However, our study also revealed significant reduction of the serum levels of vitamin A in patients with chronic liver cGVHD persisting for more than 1 month. Although there have been many studies evaluating ocular manifestations in patients with cGVHD, the present study is, to our knowledge, the first to study the relationship between vitamin A and ocular manifestations of cGVHD in humans (23). Ocular disorders commonly accompany vitamin A deficiency in infants and children, but only a few reports have indicated that vitamin A deficiency could lead to ocular changes in adults. Such ocular changes may be associated with strong T cell influx and tissue damage of the liver under vitamin A-deficient conditions (24). Decreased vitamin A levels in patients with cGVHD may increase the odds of ocular rejection through the disruption of corneal epithelial cell RNA and glycoprotein synthesis.

It is more likely that the reduction in vitamin A level is due to abnormal metabolism by the liver suffering cGVHD. Since the patients with liver cGVHD did not experience significant weight loss or gastrointestinal GVHD, vitamin A absorption were not if any impaired in these patients. It is also possible that decreased vitamin A level was secondary to increased vitamin A consumption following repair process of ocular lesions.

The goal of this report is to focus ophthalmologists’ attention on a commonly encountered problem. They should be able to recognize early symptoms and signs of severe ocular surface disorder and to think about vitamin A deficiency in particular in patients affected by cGVHD. Our study is limited in that the patients with cGVHD were also used various immunosuppressant medications that could potentially affect the levels of serum vitamin A (25). However, we believe this study point out the possible relationship between vitamin A and cGVHD.

In summary, we found that vitamin A levels were associated with the severity of ocular manifestations in patients with cGVHD. These results suggest that vitamin A is involved in the pathogenesis of ocular manifestations associated with cGVHD. Drugs aimed at increase vitamin A levels on the ocular surface in patients with cGVHD are in the pipeline, and further studies on the role of vitamin A in ocular manifestations of cGVHD will provide new strategies for the treatment of severe ocular manifestations of cGVHD.

## Ethics Statement

This study was approved by the Ethics Committee of the Second Affiliated Hospital of the School of Medicine at Zhejiang University in China, complied with the tenets of the Declaration of Helsinki for Research Involving Human Tissue, and adhered to the ARVO statement on human subjects. Written informed consent was obtained from all patients after they received an explanation of the nature and possible consequences of the procedures.

## Author Contributions

JT and RH wrote the main manuscript text. YZ, JT, and RH collected and detected the serum VitA. XJ, YZ, and RH performed ocular examination. YZ and RH prepared Tables 1–5. JT and YX diagnosed and treatment the chronic GVHD. XZ provided advice on the discussion. XJ designed and provided advice on the discussion. All authors have reviewed the manuscript.

## Conflict of Interest Statement

The authors declare that the research was conducted in the absence of any commercial or financial relationships that could be construed as a potential conflict of interest.
